# Biallelic variants in HTRA2 cause 3-methylglutaconic aciduria mitochondrial disorder: case report and literature review

**DOI:** 10.3389/fgene.2023.1298574

**Published:** 2024-01-18

**Authors:** Umamaheswaran Gurusamy, Swetha Ramadesikan, Mohammad Marhabaie, Caitlyn M. Colwell, Jesse M. Hunter, Marco L. Leung, Elaine R. Mardis, Peter White, Murugu Manickam, Richard K. Wilson, Daniel C. Koboldt

**Affiliations:** ^1^ The Steve and Cindy Rasmussen Institute for Genomic Medicine, Nationwide Children’s Hospital, Columbus, OH, United States; ^2^ Department of Pediatrics, College of Medicine, The Ohio State University, Columbus, OH, United States; ^3^ Department of Pathology, Wexner Medical Center, The Ohio State University, Columbus, OH, United States; ^4^ Division of Genetics and Genomics, Nationwide Children’s Hospital, Columbus, OH, United States

**Keywords:** leigh syndrome, HtrA2, mitochondrial disease, trio-whole exome sequencing, compound heterozygous, MGCA8, 3-MGA, autosomal recessive inheritance

## Abstract

**Background:** Leigh syndrome is a rare, genetic, and severe mitochondrial disorder characterized by neuromuscular issues (ataxia, seizure, hypotonia, developmental delay, dystonia) and ocular abnormalities (nystagmus, atrophy, strabismus, ptosis). It is caused by pathogenic variants in either mitochondrial or nuclear DNA genes, with an estimated incidence rate of 1 per 40,000 live births.

**Case presentation:** Herein, we present an infant male with nystagmus, hypotonia, and developmental delay who carried a clinical diagnosis of Leigh-like syndrome. Cerebral magnetic resonance imaging changes further supported the clinical evidence of an underlying mitochondrial disorder, but extensive diagnostic testing was negative. Trio exome sequencing under a research protocol uncovered compound-heterozygous missense variants in the *HTRA2* gene (MIM: #606441): NM_013247.5:c.1037A>T:(p.Glu346Val) (maternal) and NM_013247.5:c.1172T>A:(p.Val391Glu) (paternal). Both variants are absent from public databases, making them extremely rare in the population. The maternal variant is adjacent to an exon-intron boundary and predicted to disrupt splicing, while the paternal variant alters a highly conserved amino acid and is predicted to be damaging by nearly all *in silico* tools. Biallelic variants in *HTRA2* cause 3-methylglutaconic aciduria, type VIII (MGCA8), an extremely rare autosomal recessive disorder with fewer than ten families reported to date. Variant interpretation is challenging given the paucity of known disease-causing variants, and indeed we assess both paternal and maternal variants as Variants of Uncertain Significance under current American College of Medical Genetics guidelines. However, based on the inheritance pattern, suggestive evidence of pathogenicity, and significant clinical correlation with other reported MGCA8 patients, the clinical care team considers this a diagnostic result.

**Conclusion:** Our findings ended the diagnostic odyssey for this family and provide further insights into the genetic and clinical spectrum of this critically under-studied disorder.

## Introduction

Leigh syndrome (OMIM: #256000) is a rare and severe autosomal recessive mitochondrial disease characterized by the progressive deterioration of the central nervous system (Rahman, 2023). This disorder, also called subacute necrotizing encephalomyelopathy, was first reported by British neuropathologist Denis A. Leigh in 1951 (Leigh, 1951). In general, the estimated incidence rate of the disease is 1 per 40,000 newborn individuals worldwide (Darin, 2001); however, there are regions with a higher incidence, such as the Saguenay Lac-Saint-Jean region of Quebec, Canada (1 in 2000 newborns) and the Faroe Islands located between Iceland and Scotland (1 in 1700 newborns). Symptoms include global developmental delay, failure to thrive, ataxia, seizure, respiratory failure, hypotonia, dystonia, and ophthalmologic abnormalities, such as nystagmus, optic atrophy, ptosis, and strabismus (Rahman et al., 1996; Finsterer, 2008). Aside from these prevalent features, problems in other organ systems (cardiac and gastrointestinal) have also been reported in some cases (Finsterer, 2008). Clinical symptoms usually manifest in infancy between three and 24 months of age but later-onset forms of disease have also been described (Rahman et al., 2023).

Leigh syndrome usually manifests from a defect in mitochondrial energy production caused by deleterious changes in genes encoding enzymes of the mitochondrial respiratory chain or pyruvate dehydrogenase complex. Perhaps surprisingly, early studies indicated that many such genes are encoded by the nuclear genome (Miranda et al., 1989). Decades of research have revealed that this complex neurodegenerative disease comprises more than 100 distinct monogenic disorders with substantial clinical heterogeneity (Rahman et al., 1996). These include maternally inherited mitochondrial genes such as *ND1-6, COX III, ATPase tRNA,* and nuclear genome encoded genes including *NDUFV1-2, NDUFS1-4, 7, 8, SDHA, SDH, COX15, SURF1, CoQ, PDSS2, PDHX1, LRP130,* and *PDHc* (reviewed in Finsterer, 2008). Among them, *SURF1* (SURF1 Cytochrome C Oxidase Assembly Factor), *LRP130* (leukin-rich protein 130), and *MT-ATP6* (Mitochondrially Encoded ATP Synthase Membrane Subunit) are the most frequently mutated genes.

Ten subgroups of methylglutaconic aciduria (Type I, II, III, IV, V, VI, VIIA, VIIB, VIII, and IX) have been identified to date (Gorman et al, 2016b). Type VIII (abbreviated MGCA8 by OMIM), associated with the *HTRA2* gene (MIM: #606441), is one of the least-studied forms (Mandel et al., 2016). Only twelve MGCA8 patients from seven unrelated families have been reported in the literature as of 2023. (Mandel et al., 2016; Olahova et al., 2017; Kovacs-Nagy et al., 2018; Sreedhara et al., 2020). The paucity of genetic and phenotypic data makes diagnosis of this disorder especially challenging. Here, we describe a two-year-old male proband who demonstrated the clinical and molecular characteristics of Leigh-like syndrome. Despite extensive genetic and biochemical testing, the cause of his disorder remained elusive. The proband and his parents were consented for further studies under an IRB-approved research protocol at our hospital. Exome sequencing revealed that the proband was compound heterozygous for two novel variants in the *HTRA2* gene, conferring a diagnosis of 3-methylglutaconic aciduria type VIII (MGCA8). These findings not only ended a long diagnostic odyssey for the family, but also shed crucial new light into the variable clinical manifestations of this ultra-rare disorder.

## Case description

### Clinical characteristics

Our patient was an infant male (now deceased) clinically diagnosed with mitochondrial complex I deficiency who exhibited classic Leigh syndrome-like clinical features (Table 3). He was the first child of a non-consanguineous healthy Dutch couple. He was born at 38 weeks via Cesarean section with a length of 19 inches and a weight of 7 pounds, 14 ounces. The proband had no siblings, and there were no similar symptoms among the parents and other family members. The clinical manifestations include nystagmus (HP: 0000639), hypotonia (HP: 0001252), respiratory failure (HP: 0002878), stridor (HP: 0010307), loss of weight (HP: 0001824), brain imaging abnormality (HP: 0410263), feeding difficulties (HP:0011968) and developmental delay (HP: 0002194). The initial differential diagnosis included metabolic disease, lysosomal storage disorder, and Juvenile Huntington’s disease. Ultimately, he was given a clinical diagnosis of Leigh-like syndrome based on his clinical manifestations and progression of features.

### Diagnostic testing

The patient’s clinical workup began when he was 6 months of age. An EEG was normal. A heart echo and EKG showed a small ventricular septal defect (VSD) but were otherwise normal. Cerebral magnetic resonance imaging (MRI) findings showed multifocal areas of new and old symmetric deep gray matter injury involving the thalamus, caudate, and globus pallidus bilaterally; MR spectroscopy indicated a possible lactate peak consistent with the clinical diagnosis of Leigh syndrome. Urine organic acid testing revealed prominent medium chain dicarboxylic aciduria with 7-OH octanoic and 3-OH-sebacic (considered the result of using medium-chain triglyceride supplemented formula) but was otherwise normal. Other studies–creatine, calcium, CK enzyme, cholesterol, glucose, sed rate, plasmy acylcarnitine profile, serum amino acids, thyroid function studies, biotinidase, and long chain fatty acids–were normal.

A muscle biopsy was performed under general anesthesia at 9 months of age. Electron transport chain enzyme testing revealed normal citrate synthase activity, partial reduction in complex II + III and rotenone sensitive I/III activities, and a deficiency in complex I (NADH dehydrogenase). The complex I deficiency suggested a respiratory chain disorder, but comprehensive mitochondrial testing by next-generation sequencing (Baylor) was nondiagnostic. Mitochondrial DNA screening, karyotyping, single nucleotide polymorphism microarray, and *POLG1* testing were performed but were nondiagnostic. The patient and both parents were then consented for an IRB-approved research study for further genomic testing.

### Technical analysis and methods

In compliance with the Declaration of Helsinki, the study was conducted with the approval of the Institutional Review Board (IRB) of the Nationwide Children’s Hospital (NCH). After obtaining written informed consent from the parents, peripheral venous blood was collected from the family trio. We first extracted the genomic DNA from the peripheral leucocytes of the child and parents using the NEBNext Ultra II FS DNA Library Prep Kit (New England BioLabs) according to the manufacturer’s instructions. Whole exome sequencing was performed at the Steve and Cindy Rasmussen Institute for Genomic Medicine. Exome capture was performed using the IDT xGen capture kit with CNV spike-in, and sequencing was performed on a NovaSeq6000 instrument (Illumina Inc., San Diego, CA, United States). Bioinformatics processing and variant analysis were conducted as described previously (Koboldt et al., 2018). Briefly, all sequencing data were mapped with the human reference genome (GRCh38). After recalibration and de-duplication of the reads, we proceeded with the variant calling and analysis using the Churchill framework (Kelly et al., 2015). We generated 12-13 Gbp of data per individual achieving ∼82x average target depth, with >96% of the exome covered >20x. Detailed sequencing metrics are provided in Supplementary Table S1. The variant annotation, classification, and prioritization were performed according to our standard approach for rare diseases, which has been described previously (Koboldt et al., 2018). Generally speaking, we selected rare damaging variants that segregated under dominant (*de novo*), recessive, or X-linked inheritance models and used data from OMIM, ClinVar, and other public databases to prioritize candidate genes (Koboldt et al., 2018). Variants were confirmed by Sanger sequencing of PCR-amplified products from the proband and negative control reference samples using BigDye Terminator cycle sequencing chemistry (Applied Biosystems) on an ABI 3730xl Genetic Analyzer.

### Variant detection and diagnostic assessment

**FIGURE 1:**
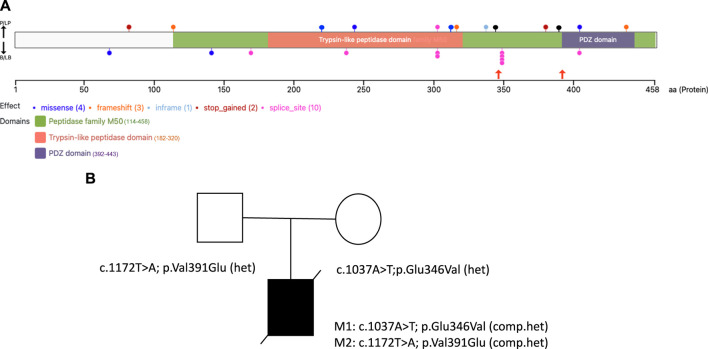
HTRA2 protein structure with disease-causing variants and the family pedigree of the proband **(A)**. The VanDyPlot v0.1.1 depicts the ClinVar database pathogenic variants plotted on the HTRA2 protein (UniProt ID: 043464). Variants are shown at their predicted protein position as colored circles reflecting the effect type, with the two missense variants (black lollipops) reported here shown in orange arrows, and the two blue lollipops at position 221 and 315 were the missense variants reported from the literature. The HTRA2 protein domain annotations are retrieved from Pfam database of curated protein families **(B)**. Family pedigree showing the inheritance of two pathogenic compound heterozygous variants of the *HTRA2* gene in the proband.

The proband was compound heterozygous for two novel missense variants in the *HTRA2* gene (transcript ID ref: NM_013247.5; see Figure 1B) located on chromosome 2p13.1. The maternally-inherited variant (c.1037A>T in exon 5) is absent from public databases (gnomADv3), making it extremely rare. The predicted amino acid change (p.Glu346Val) is benign by most computational tools (REVEL = 0.145). However, this variant creates a GT dinucleotide 8 bp upstream of the exon-intron boundary and is predicted by multiple computational tools to create a novel splice donor site (SpliceAI = 0.85, Pangolin = 0.76). Patient RNA was not available to confirm disrupted splicing. The paternally-inherited variant (c.1172T>A in exon 7) is also absent from public databases (gnomADv3). This amino acid change (p.Val391Glu) is predicted to be damaging by virtually all *in silico* tools including meta-predictors (REVEL = 0.868, MetaRNN = 0.9511). There were no alternate candidate genes or significant variants identified in our investigation.

Under current ACMG guidelines (Richards et al., 2015), both *HTRA2* variants are classified as Variants of Uncertain Significance (Table 1), but given the significant clinical correlation, the patient care team considers this a diagnostic result. Both variants were independently validated by Sanger sequencing in a CLIA/CAP certified clinical laboratory.

**TABLE 1:** Genomic findings and variant interpretation. Variant interpretation was performed using ACMG 2015 guidelines (Richards et al., 2015). Interpretation evidence codes include PM2 (rare or absent in populations), PP3 (computational studies show deleterious effect). For the maternal variant, evidence code PP3 is applied due to the variant’s proximity to the intron-exon boundary and a computationally predicted effect on splicing (SpliceAI score = 0.85, Pangolin score = 0.76). For the paternal variant, evidence code PP3 is applied based on computational predictions of damaging effect for the amino acid change (REVEL = 0.868, MetaRNN = 0.9511). We considered but did not apply PP4 (phenotype highly specific for gene) given the paucity of data about HTRA2-associated disease.

Gene	Variant (GRCh38)	Location	Inheritance	Amino acid change	Interpretation
*HTRA2*	2-74531694-A-T	Exon 5	Maternal	(p.Glu346Val)	VUS (PM2, PP3)
*HTRA2*	2-74532675-T-A	Exon 7	Paternal	(p.Val391Glu)	VUS (PM2, PP3)

At present, the ClinVar database (https://www.ncbi.nlm.nih.gov/clinvar) contains 9 pathogenic/likely pathogenic variants in *HTRA2* (excluding large events and structural variants), including 6 truncating variants (3 frameshift, 2 nonsense, 1 splice site) and 3 nonsynonymous variants (2 missense, 1 inframe indel), see Figure 1A. The relatively small number of disease-causing variants reported for this gene makes variant interpretation challenging.

### Genotype and phenotype spectrum of HTRA2 deficient MGCA8

The very first case of deleterious *HTRA2* recessive variants was identified by Mandel et al., in 2007. They studied four affected infants from two unrelated families (family A: Druze origin; family B: Ashkenazi-Jewish origin) segregating autosomal inheritance MGCA8 and found linkage to the *HTRA2* locus; genetic analysis of this gene revealed two pathogenic compound heterozygous (a splice-disruptive mutation and a deletion). The patients presented with lactic acidemia, evolving brain atrophy, disturbed cristae structure in muscle mitochondria 3-MGA. Subsequent functional studies of the patient fibroblasts revealed HTRA2 is essential to brain development, and its absence due to recessive deleterious mutations result in MGCA8, increased sensitivity to apoptosis, abnormal mitochondria, and infantile neurodegeneration (Mandel et al., 2016). This outcome was echoed in a study by Olahova and others (Olahova et al., 2017) from the United Kingdom in five affected individuals from two unrelated families (Pakistan and Mexican origin). The proband symptoms include seizures, neutropenia, hypotonia, feeding difficulties, central apneas, and cardio-respiratory problems. They identified two biallelic homozygous variants, a splice site variant c.906 + 1G>C (p.Gly261_Asp302del, Asp302_Phe303ins*35) and c.728_730delinsCAT (p.Leu243_Pro244delinsProSer) in the proband of Pakistan and Mexican origin, respectively (Olahova et al., 2017). In addition, they observed a complete loss of HTRA2 protein in the homogenates of the patient’s fibroblasts and skeletal muscle. Similarly, in another investigation, Kovacs-Nagy and co-workers reviewed the clinical features of two patients with MGCA8 and found two rare variants c.346insG; p. Ala116Glyfs*22 and c.944G>A, p. Gly315Glu in the French individual. The first variant is a frameshift mutation predicted to cause a premature stop codon, while the other variant is predicted to disturb the highly conserved amino acid. The Iraqi patient harbored a homozygous variant c.1013_1015del; p. (Leu338del), which results in the deletion of an evolutionally conserved amino acid. Both patients presented with recognizable phenotypes consistent with the previously reported cases (Kovacs-Nagy et al., 2018).

We compiled all the phenotype features and the relevant HPO terms of patients from eight independent families and established the overall percentage of each phenotype in this series (Table 2). In thirteen patients with MGCA8, 92.3% had feeding difficulties (HP:0011968), 61.5% had respiratory failure (HP:0002878), 53.8% had tremor (HP:0001337), and hypotonia (HP:0001252) was detected in 69.2% of the patients. We also collected clinical summaries, metabolic investigations, radiological results, and genetic findings of all reported MGCA8 patients including ours (Table 3). The overall clinical picture of this disease is characteristic of Leigh syndrome: neonatal-onset symptoms (feeding difficulties, poor weight gain), progressive neurodevelopmental delay/regression, brain lesions, biochemical abnormalities, and early lethality. Most published MGCA8 patients also shared a unifying biochemical phenotype, 3-Methylglutaconic aciduria, a biomarker of mitochondrial dysfunction. This biochemical signature was not apparent in our proband when he underwent urine organic acid testing at 7.5 months of age.

**TABLE 2:** Overview of the HPO terms related to 3-methylglutaconic aciduria in our patient and from previous reports.

HPO terms	Features	Dutch^a^ (N = 1)	Indian^a^ (N = 1)	Iraqi^b^ (N = 1)	French^b^ (N = 1)	Mexican^c^ (N = 3)			Pakistani^c^ (N = 2)		Ashkenazi^d^ (N = 1)	Druze^d^ (N = 3)			All % (N = 13)
HP:0000007	Autosomal recessive inheritance	+	+	+	+	+	+	+	+	+	+	+	+	+	100
HP:0000639	Nystagmus	+													7.7
HP:0001252	Hypotonia	+	+	+	+				+		+	+	+	+	69.2
HP:0002878	Respiratory failure	+	+		+	+	+		+	+		+			75.0
HP:0010307	Stridor	+													7.7
HP:0002169	Clonus		+	+	+			+							30.8
HP:0001824	Loss of weight	+					+		+						23.1
HP:0001263	Developmental delay	+									+	+	+	+	38.5
HP:0012332	Dysautonomia				+						+				15.4
HP:0001662	Bradycardia							+			+				15.4
HP:0001250	Seizure/Epilepsy			+		+	+					+	+	+	46.1
HP:0001332	Dystonia			+					+			+	+	+	38.5
HP:0003202	Skeletal muscle atrophy					+									7.7
HP:0012416	Hypercarbia					+									7.7
HP:0001643	Patent ductus arteriosus					+									7.7
HP:0003316	Butterfly vertebrae										+				7.7
HP:0410263	Brain Image abnormality	+				+					+		+	+	38.5
HP:0001276	Hypertonia		+		+							+	+	+	38.5
HP:0000407	Sensorineural deafness												+	+	15.4
HP:0001298	Encephalopathy										+				7.7
HP:0011968	Feeding difficulties/problems	+	+	+	+	+	+		+	+	+	+	+	+	87.5
HP:0002015	Dysphagia			+	+				+						23.1
HP:0002104	Apnea		+	+	+				+		+	+			46.1
HP:0040213	Hypopnea			+	+	+									23.1
HP:0001875	Neutropenia		+	+					+		+	+	+	+	53.8
HP:0001903	Anemia			+											7.7
HP:0000252	Microcephaly			+							+				15.4
HP:0001337	Tremor		+	+				+			+	+	+	+	53.8
HP:0000518	Cataracts								+						7.7
HP:0002151	Increased serum lactate		+								+	+	+	+	38.5
HP:0012707	Elevated brain lactate level by MRS		+		+	+					+				30.8
HP:0003128	Lactic acidosis			+											7.7
HP:0005972	Respiratory acidosis		+												7.7
HP:0012087	Abnormal mitochondrial shape													+	7.7
HP:0000952	Jaundice						+								7.7
HP:0001943	Hypoglycemia								+						7.7
HP:0003344	3-methylglutaric acid			+	+	+	+	+				+	+	+	61.5
HP:0003535	3-methylglutaconic acid		+	+	+	+	+	+	+	+	+	+	+	+	92.3

HPO, Human Phenotype Ontology, N–number of subjects.

^a^ This study.

^a^ Sreedhara et al. (2020).

^b^ Kovacs-Nagy et al. (2018).

^c^ Olahova et al. (2017).

^d^ Mandel et al. (2016).

**TABLE 3:** Summary of clinical, biochemical, and molecular findings in 3-MGA patients with *HTRA2* mutations.

Study	Family origin	Age at diagnosis	Gender	Urine 3-MGA levels	Age at death	Clinical and biochemical features	*HTRA2* Variant(s)
Mandel et al. (2016) ^*^	Druze Patient 1	newborn	f	elevated	10 days	tremor, feeding difficulties, hypertonia, developmental delay, hypotonia, neutropenia, intractable seizure, apneic spell, dystonic posturing	c.1211 G>A (p.Arg404Gln; p.R404Q)
	Patient 2	newborn	f	elevated	10 months	feeding difficulties, developmental day, carpus callosum, neutropenia, seizure, hypertonia, hypotonia, tremor, dystonic movements, sensorineural deafness	c.1211 G>A (p.Arg404Gln; p.R404Q)
	Patient 3	newborn	m	elevated	10 months	abnormal mitochondrial shape, hypertonia, hypotonia, seizure, developmental delay, tremor, dystonic movements, feeding difficulties, sensorineural deafness, neutropenia, carpus callosum	c.1211 G>A (p.Arg404Gln; p.R404Q)
	Ashkenazi-Jewish	newborn	m	elevated	5 months	poor feeding, hypotonia, recurrent apnea, tremor, neutropenia, dysautonomia, cerebral volume loss, butterfly shaped T8 vertebrate, bradycardia, poor muscle tone, jerks, elevated lactate, sagittal sinus venous thrombosis, microcephaly, myoclonic encephalopathy, developmental delay	c.1312_1316del; (p.Ala438fs)
Olahova et al. (2017) ^*^	Pakistan S1	newborn	m	elevated	2 months	hypoglycemia, hypotonia, dysphagia, recurrent apnea, weight loss, poor feeding, respiratory failure, dystonic limb movements, bilateral cataracts, neutropenia, poor cardiac contractility	c.906 + 1G>C; (p.Gly261_Asp302del, Asp302_Phe303ins^b^35)
	S1-sibling	newborn	m	n/a	3 weeks	feeding difficulties, breathing problem, recurrent apnoea, dysphagia	n/a
	Mexican S2	newborn	m	elevated	3 months	hypercarbia, central hypopnea, seizure, muscle denervation and atrophy, patent ductus arteriosus, respiratory insufficiency, feeding difficulties, elevated lactate and lipid peaks on MRS	c.728_730delinsCAT; (p.Leu243_Pro244delinsProSer)
	S3	newborn	f	elevated	4 months	poor feeding, weight loss, respiratory failure, seizure, jaundice	n/a
	S4	newborn	m	elevated	3 months	bradycardia, tremor, clonus of both lower extremities	n/a
Kovacs-Nagy et al. (2018) ^*^	French	newborn	m	elevated	43 days	respiratory and multiorgan failure, peripheral muscular hypertonia, clonic limb movements, dysautonomia, feeding difficulties, vomiting, diarrhea	c.346insG; (p.Ala116Glyfs^b^22) c.944G>A; (p.Gly315Glu)
	Iraqi	newborn	f	elevated	64 days	microcephaly, apnea, generalized muscular hypotonia, therapy-resistant epileptic seizure, anemia, neutropenia, tremor, jitteriness, lactic acidosis, respiratory insufficiency, apnea, feeding difficulties	c.1013_1015del; (p.Leu338del)
Sreedhara et al. (2020)	Indian	newborn, 7 days	f	borderline elevation	3 months	truncal hypotonia, axial hypertonia, ankle clonus, encephalopathy, respiratory distress, feeding difficulty, apnea, neutropenia, depressed sensorium, respiratory acidosis, elevated lactate, stimulus-sensitive tremulousness	c.661A>C, (p.Thr221Pro)
This study (2023)^*^	Dutch		m	normal	14 months	nystagmus, hypotonia, developmental delay, respiratory failure, stridor, loss of weight, feeding difficulties, brain image abnormality	c.1037A > T; (p.Glu346Val) c.1172T > A; (p.Val391Glu)

^a^ -MGA, 3-methylglutaconic aciduria; f, female; m, male; MRS, magnetic resonance spectroscopy.

^b^ Whole Exome Sequencing study; n/a, not available. S, subject.

Overall, the relatively few MGCA8 patients described in the literature exhibit considerable phenotypic heterogeneity. The expert clinicians who cared for our patient considered the overall presentation consistent with a diagnosis of MGCA8 even without definitive proof of the classic biochemical finding.

## Discussion

Mitochondrial diseases are the most heterogenous and commonly inherited metabolic disorders, often presenting with a broad spectrum of clinical manifestations (Gorman et al., 2016a). One such disorder, 3-methylglutaconic aciduria type VIII (MGCA8, MIM: #617248), is a rare, heritable, autosomal recessive inborn error of metabolism associated with variants in the *HTRA2* gene. Human HTRA2 is a 49 kDa nuclear-encoded mitochondrial protein composed of 458 amino acids. It belongs to the HTRA family of stress-induced serine proteases (Mandel et al., 2016) and it is essential for maintaining mitochondrial homeostasis. *HTRA2* is also involved in apoptosis via caspase-dependent and independent pathways (Simón-Sánchez and Singleton, 2008; Mandel et al., 2016). Yet the precise mechanism by which HTRA2 deficiency causes mitochondrial disease remains unclear.

Among the relatively small number of MGCA8 patients reported in the literature to date, core clinical features include hypotonia, seizure, abnormal movements, encephalopathy with depressed sensorium, respiratory insufficiency, and developmental delay. The proband report here shared many of these features. However, he did not exhibit the markedly elevated 3-methylglutaconic and 3-methylglutaric acids in urine, which until recently was considered a unifying feature of this clinically variable, ultra-rare disorder (Olahova et al., 2017; Kovacs-Nagy et al., 2018), was also a unifying feature. However, we note that our patient survived longer (10 months) and underwent urine organic acid testing later (7.5 months of age) than all previously reported patients. Furthermore, a recently published MGCA8 patient from India exhibited the common clinical manifestations but showed only borderline elevated levels of 3-MGA (Sreedhara et al., 2020). It is therefore possible that this biochemical signature is common but not diagnostic for HTRA2 deficiency, similar to other forms of genetic 3-methylglutaconic aciduria such as Barth syndrome and 3-MGA2 (MIM: #302060) (Cantlay et al., 1999; Schmidt et al., 2004). Because our patient passed away before the genetic diagnosis was made, we were unable to repeat urine organic testing to confirm the absence of 3-MGA. In any case, the preponderance of evidence including the genetic findings, consistent clinical features, and rapid progression of symptoms in our patient support the finding that MGCA8 is the diagnosis.

## Conclusion

In summary, we used research WES to identify compound-heterozygous missense variants in *HTRA2* in a patient with Leigh-like mitochondrial disease who had extensive negative prior testing. Our findings end a long diagnostic odyssey for the patient family and shed further light on the clinical and genetic spectra of this ultra-rare recessive disorder. Although extremely rare, MGCA8 has proven a severe and consistently lethal disease, underscoring the need for rapid genomic testing in symptomatic patients with or without 3-MGA.

## Data Availability

The datasets presented in this study can be found in online repositories. The names of the repository/repositories and accession number(s) can be found below: https://www.ncbi.nlm.nih.gov/clinvar/submitters/506343/, ClinVar: SCV002058111.1.
